# MicroRNA analysis of gastroenteropancreatic neuroendocrine tumors and metastases

**DOI:** 10.18632/oncotarget.25357

**Published:** 2018-06-19

**Authors:** Nadine Zimmermann, Juliana Knief, Tim Kacprowski, Pamela Lazar-Karsten, Tobias Keck, Franck Billmann, Sebastian Schmid, Kim Luley, Hendrik Lehnert, Georg Brabant, Christoph Thorns

**Affiliations:** ^1^ Department of Pathology, Section of Hematopathology and Endocrine Pathology, University Hospital of Schleswig-Holstein, Lübeck 23538, Germany; ^2^ Department of Functional Genomics, Interfaculty Institute of Genetics and Functional Genomics, University Medicine Greifswald, Greifswald 17475, Germany; ^3^ Department of Surgery, University Hospital of Schleswig-Holstein, Lübeck 23538, Germany; ^4^ Department of Internal Medicine 1, University Hospital of Schleswig-Holstein, Lübeck 23538, Germany

**Keywords:** gastroenteropancreatic tumors, neuroendocrine neoplasias, microRNA, metastatic disease

## Abstract

The incidence of neuroendocrine neoplasias (NEN) continues to increase. Since the primary tumor cannot be diagnosed in some cases of metastatic disease, new biomarkers are clearly needed to find the most probable site of origin. Tissue samples from 79 patients were analyzed and microRNA profiles were generated from a total of 76 primary tumors, 31 lymph node and 14 solid organ metastases. NEN metastases were associated with elevated levels of miR-30a-5p, miR-210, miR-339-3p, miR-345 and miR-660. Three microRNAs showed a strong correlation between proliferation index and metastatic disease in general (miR-150, miR-21 and miR-660). Further, each anatomic location (primary or metastatic) had one or more site-specific microRNAs more highly expressed in these tissues. Comparison between primary tumors and metastases revealed an overlap only in pancreatic (miR-127) and ileal tumors (let-7g, miR-200a and miR-331). This thorough analysis of gastroenteropancreatic neuroendocrine tumors demonstrates site-specific microRNA profiles, correlation with proliferation indices as well as corresponding nodal and distant metastases. Using microRNA profiling might improve NEN diagnostics by linking metastases to a most probable site of origin.

## INTRODUCTION

Neuroendocrine neoplasias (NEN) are still rare tumors despite a growing incidence in the last decades (1.09 cases/100.000 in 1973 vs 5.25 cases/100.000 in 2004). They represent approximately 1.25% of all malignant diseases [1]. Gastroenteropancreatic neuroendocrine neoplasias (GEP-NEN) constitute 50–67% of all NEN but only 2% of all tumors in the gastrointestinal tract [2, 3]. There is an equal distribution between both sexes (1.06:1 - f: m) with a mean age of 60 years at presentation [3]. 5-year-survival rates vary considerably between 37.5% (pancreatic NEN) and 88.3% (rectal NEN) depending on location, tumor stage and patients age [2, 4, 5]. GEP-NEN are most frequently encountered in the ileum (35.5%), followed by tumors in the rectum and appendix (20% and 17.8%, respectively). However, the primary tumor cannot be detected in 0.6–2% of metastatic disease (CUP - cancer of unknown primary site) despite extensive clinical and radiographic search [4]. Even histopathologic evaluation with immunohistochemical analysis is not always helpful in determining the origin of the primary tumor. Different panels containing various antibodies (i.e. CDX2, PAX8, TTF1, Islet 1 as well as steroid hormone receptors) have been proposed but can - in a substantial proportion of cases–not differentiate reliably between primaries of different sections in the gastroenteropanceatic system [6–9].

MicroRNAs (miRs) are small, non-coding RNAs with a length of 21–25 nucleotides and participate in gene regulation on the post-transcriptional level [2, 10]. Several miRs act as either oncogenes (so-called “onco-miRs“) or tumor suppressors thereby influencing the growth and dissemination of tumors [11, 12]. Multiple studies demonstrated that miR-expression profiles could potentially be used as diagnostic and prognostic markers and probably be even an important diagnostic factor in determination of the primary site cases in CUP [4, 5, 13, 14].

Up to now, data of miR-expression profiles in GEP-NEN are still scarce: Some smaller studies showed that expression of miR-222 in gastric NEN (gNEN) type 1 (the most common type) is significantly higher compared to normal gastric tissue [15]; a high expression of miR-885-5p in rectal NEN is associated with invasion of lymphatic vessels [16]. In small bowel tumors–especially those from the ileum–39 dysregulated miRs were detected including miR-204-5p, miR-7-5p and miR-375 which all showed upregulation. Comparison of liver and lymph node metastases with primaries in the ileum showed a multitude of differentially expressed miRs including miR-1, miR-10b, miR-129-5p, miR-133a, miR-143-3p, miR-145, miR-146, miR-215, miR-222 and miR-31 (all down-regulation) as well as miR-183, miR-19a, miR-19b, miR-200a and miR-488 (all up-regulation) [17–20].

Concerning pancreatic NEN (pNEN), in one large study, 28 different miRs have been shown to be differentially expressed compared to healthy pancreatic tissue with 18 of them being higher expressed and 10 lower expressed [21]. Further studies also showed lower expression of let-7 miR and miR-155 as well as higher expression of miR-103, miR-107 and miR-193b in pNEN [22–24]. Regarding prognostic factors, expression levels of miR-196a, miR-21 and miR-642 have been shown to correlate with tumor proliferation (defined byKi67). Furthermore, miR-210 and miR-21 seem to correlate with metastatic disease [23–27] and expression of both miR-196a and miR-27b are predictive for tumor recurrence [26].

To our knowledge, no studies specifically addressed miRs in neuroendocrine neoplasias of different anatomic sites as compared to their corresponding metastases. We therefore aimed to determine unique miR-expression profiles in different anatomic sites of GEP-NEN with the overarching goal to predict the (most probable) primary tumor site from corresponding metastases. As the primary tumor often remains elusive despite extensive searching in CUP, this would clinically impact future diagnostic and therapeutic algorithms in GEP-NEN.

## RESULTS

### Clinical and pathological characteristics

We analyzed 121 GEP-NEN samples of 79 patients including 76 primary tumors (pt), 31 lymph node metastases and 14 solid organ metastases. The majority of tumors originated in the pancreas (48.68%) followed by ileum (14.47%), stomach (11.84%) and appendix (10.53%) while only 7.89%, 3.95% and 2.63% of primary tumors derived from rectum, ascending colon and caecum, respectively. Patients with primary appendix NEN were significantly younger than those with NEN at other primary sites (mean age 30.6 years vs. 52.2–67.2 years; *p*-value 0.0014). Distribution between sexes was fairly balanced for most anatomic sites, except for tumors of the ileum, ascending colon and caecum being more prevalent in men. Concerning TNM stage, half of the patients (38 cases; 50%) were diagnosed with localized disease (pT1 and pT2 categories) and more than a fifth (17 cases; 22.37%) showed no lymph node metastases. These characteristics were further reflected in (clinical) UICC staging, classifying nearly half of the cohort in UICC stages I and II (30 cases; 39.47%). A detailed overview of primary sites, distribution between sexes and age, as well as grading, TNM classification (according to the 8th edition, 2017) and UICC stage is shown in Table 1.

**Table 1:** Characteristics of GEP-NEN according to primary site, age, sex, histologic grading, TNM classification and UICC stage

primary site	stomach	pancreas	ileum	appendix	caecum	ascending colon	rectum
*N*	9	37	11	8	2	3	6
age in years (mean/ median)	61/64	52.2/51	56.3/57.5	30.6/25	59/59	62.7/60	67.2/71
sex (f:m)	1.4:1	1.4:1	1:3	1:1.7	0:2	0:3	1:1
grading G1 G2 G3	5 2 2	25 8 4	6 5 0	8 0 0	2 0 0	0 0 3	4 0 2
depth of infiltration pT1 pT2 pT3 pT4 pTX	3 0 3 1 2	16 9 8 3 1	0 3 5 3 0	7 0 1 0 0	1 0 0 0 1	0 0 1 2 0	4 0 0 2 0
nodal stage pN0 pN1 pNX	0 4 5	19 9 9	1 9 1	0 1 7	0 2 0	0 3 0	0 2 4
distant metastases M1a M1b M1c M0	3 0 0 6	4 5 1 27	2 0 1 8	0 0 1 7	0 0 1 1	0 1 0 2	0 0 0 6
UICC stage I II III IV not evaluable	3 0 2 3 1	15 6 6 10 0	0 1 7 3 0	6 1 0 1 0	0 0 1 1 0	0 0 2 1 0	4 0 2 0 0

### miR profiles according to primary sites

Analysis of miR profiles of different anatomic sites revealed that primaries in the pancreas, ileum, appendix and rectum all have at least one specific miR differentially expressed only in these tumors. The rate was highest in pNEN with 13 specific miRs, followed by ileum (9 miRs), appendix (3 miRs) and rectum (1 miR). No such unique miR-expression pattern was found for both stomach and ascending colon. A summary of differentially expressed miRs and corresponding anatomic sites is depicted in Table 2.

**Table 2:** Overview of differentially expressed miRs in relation to different primary sites

primary site	pancreas	ileum	appendix	rectum
overexpressed miRs	let-7e miR-126 miR-127 miR-30a-3p miR-409-3p miR-539 miR-652 miR-95	let-7g miR-16 miR-200a miR-320 miR-324-3p miR-331 miR-342-3p miR-744	miR-125b	miR-151-3p
down-regulated miRs	miR-155 miR-193b miR-28-3p miR-642 miR-886-5p	miR-92a	miR-200c miR-223 miR-24	

Only miRs differentially expressed in specific anatomic sites are shown.

### miR expression and correlation with metastases

**Figure 1:**
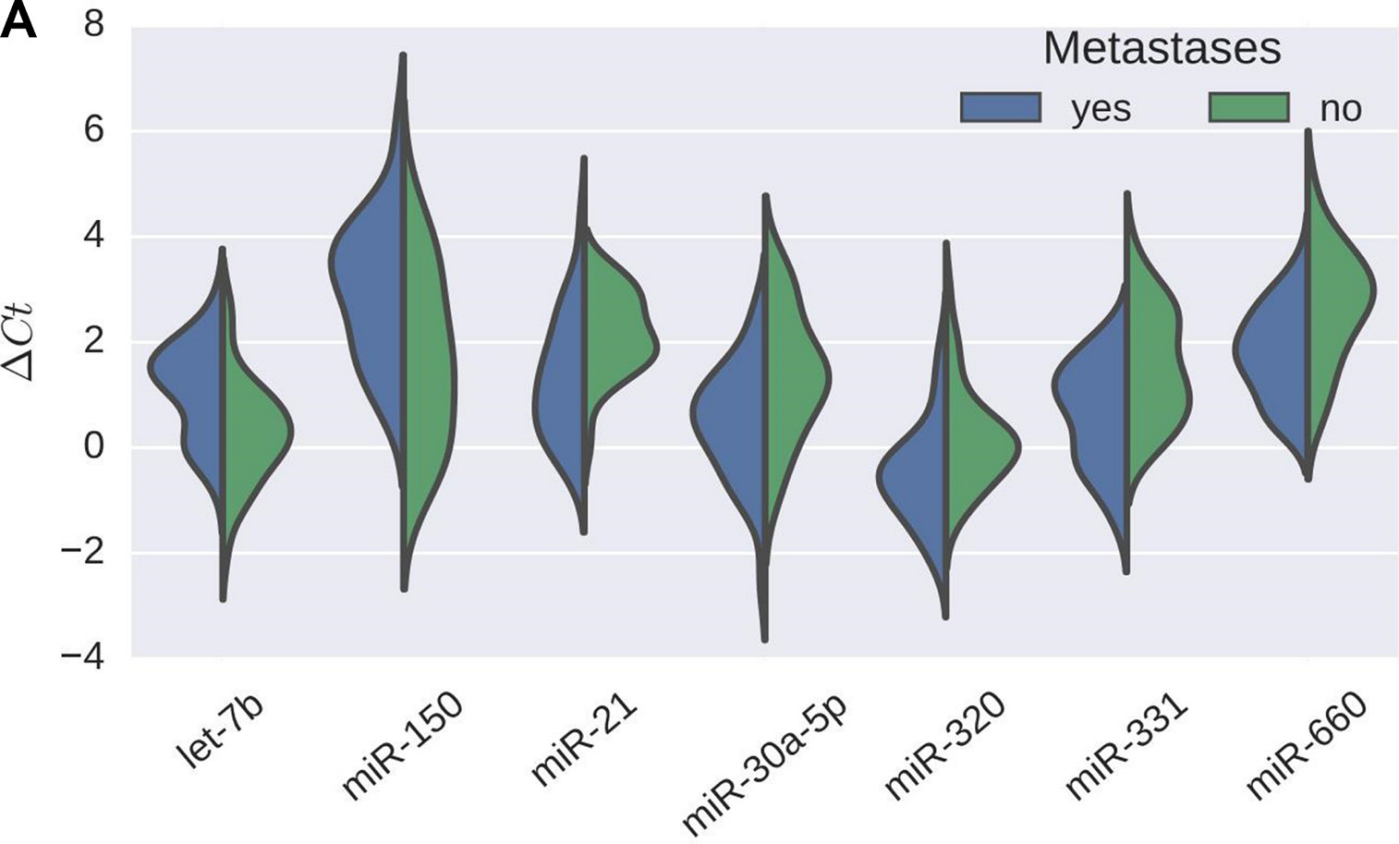

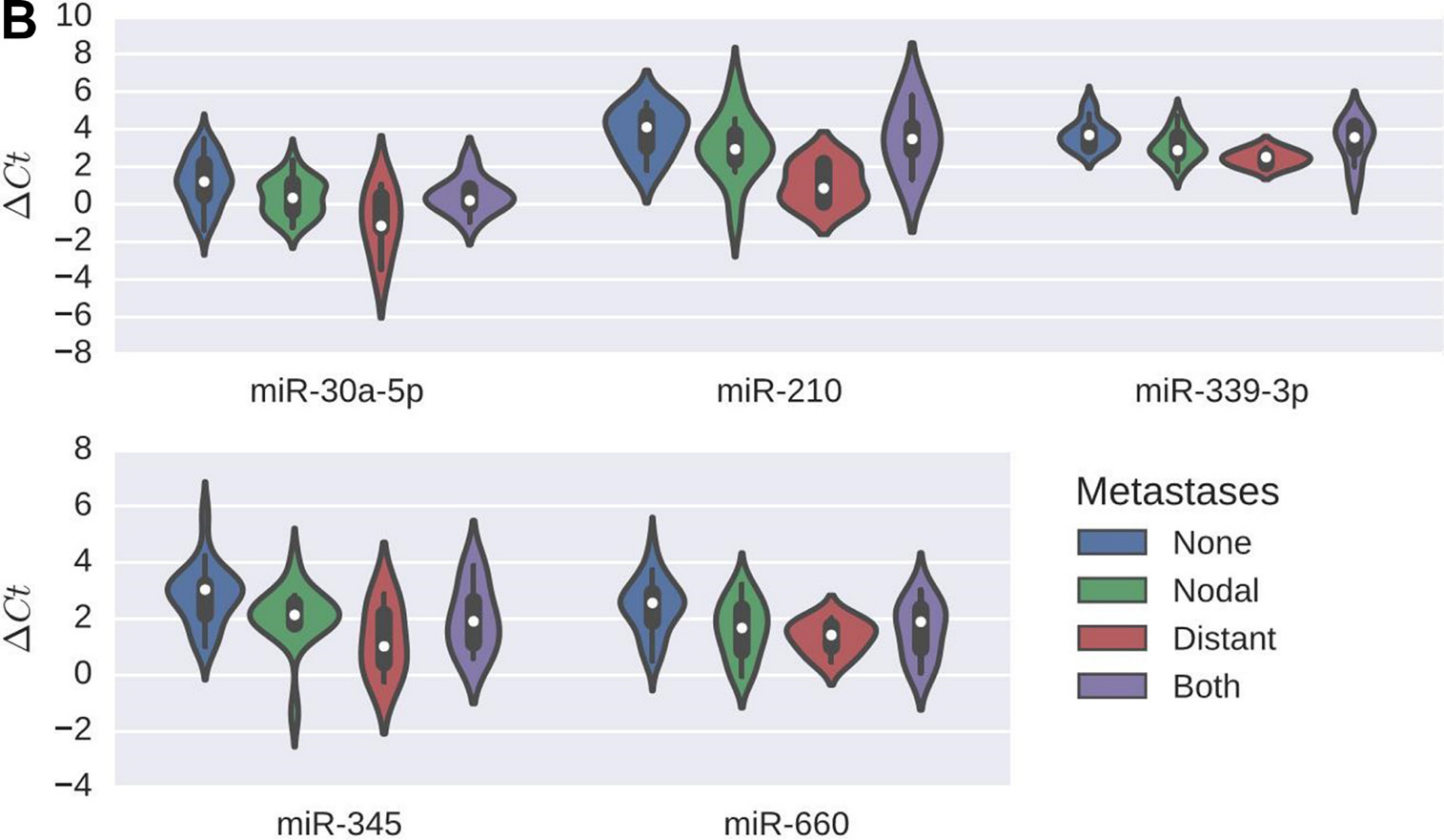

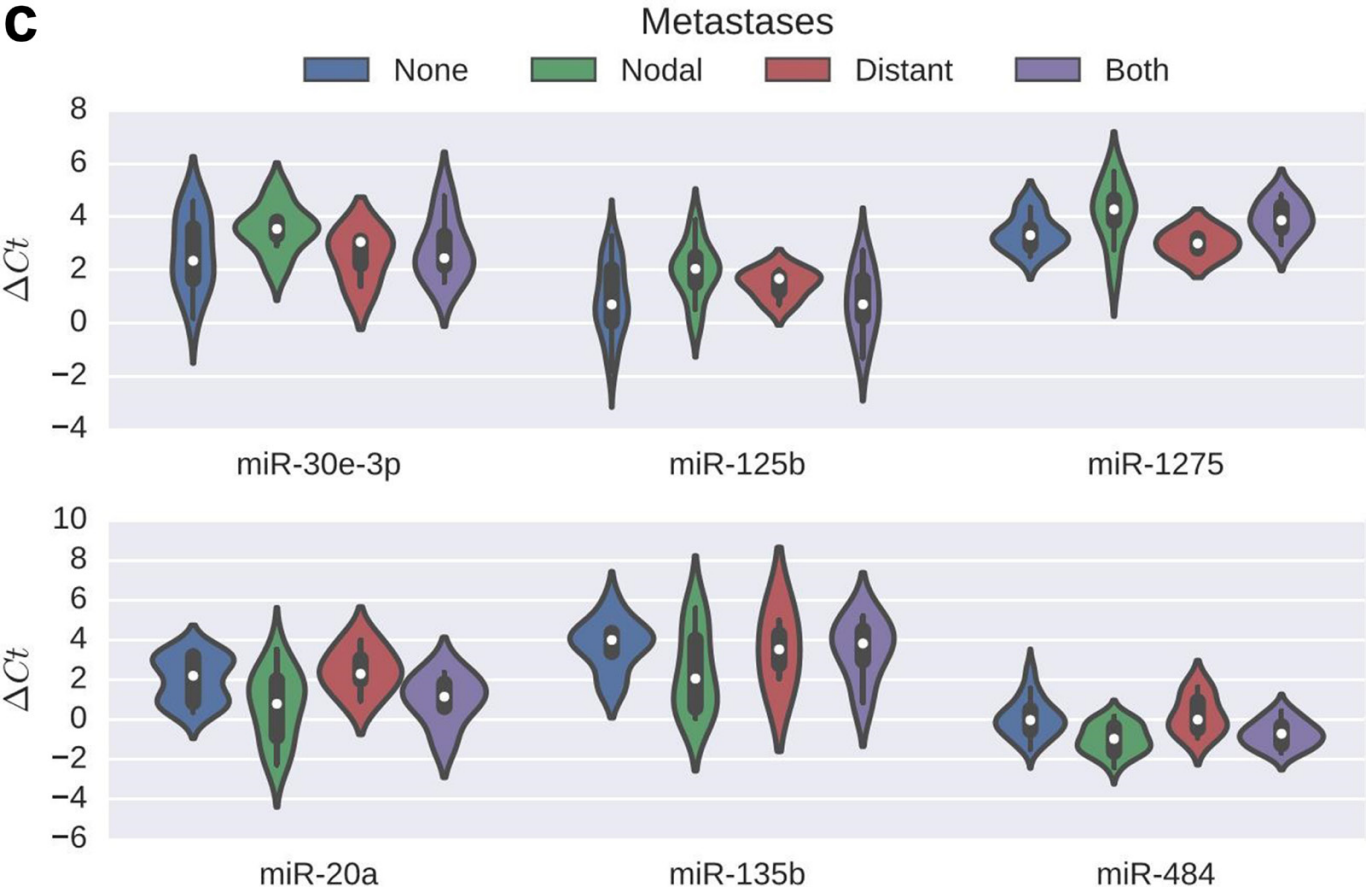
(**A**) miR-expression in cases with metastases compared to those without. Violin plots showing ∆Ct values for differentially expressed miRs. Blue part of plots represent cases with confirmed metastases, green part of plots cases without metastases. Each half of a violin depicts the distribution of ∆CT values in the respective group. Plots from left to right: ∆Ct values for let-7b (*p* = 0.022), miR-150 (*p* = 0.038), miR-21 (*p* = 0.038), miR-30a-5p (*p* = 0.038), miR-320 (*p* = 0.038), miR-331(*p* = 0.022) and miR-660 (*p* = 0.038). Lower ∆Ct values indicate higher miR expression and vice versa. (**B**) Abundance of miRs differentially expressed between cases with and without distant metastases, respectively. Violin plots showing ∆Ct values for differentially expressed miRs. Blue violin plots represent cases without metastases, green violin show cases with nodal metastases, red violin plots depict cases with distant metastases, purple violin plots represent cases with both, nodal and distant metastases. The center of each violin holds a boxplot indicating the median (white dot). Plots from left to right: Upper row: ∆Ct values for miR-30a-5p (*p* = 0.0045), miR-210 (*p* = 0.0001) and miR-339-3p (*p* = 0.0005). Lower row: ∆Ct values for miR-345 (*p* = 0.0045) andmiR-660 (*p* = 0.0127). Lower ∆Ct values indicate higher miR expression and vice versa. (**C**) Abundance of miRs differentially expressed between cases with and without nodal metastases, respectively. Violin plots showing ∆Ct values for differentially expressed miRs. Blue violin plots represent cases without metastases, green violin plots those with nodal metastases, red violin plots those with distant metastases, and purple violin plots those with both nodal and distant metastases. The center of each violin holds a boxplot indicating the median (white dot). Plots from left to right: Upper row: exemplary ∆Ct values for down-regulated miRs: miR-30e-3p (*p* = 0.00049), miR-125b (*p* = 0.013), miR-1275 (*p* < 0.0001). Lower row: exemplary ∆Ct values for overexpressed miR-20a (*p* = 0.00069), miR-135b (*p* = 0.00049), miR-484 (*p* = 0.0015). Lower ∆Ct values indicate higher miR expression and vice versa.

**Figure 2:**
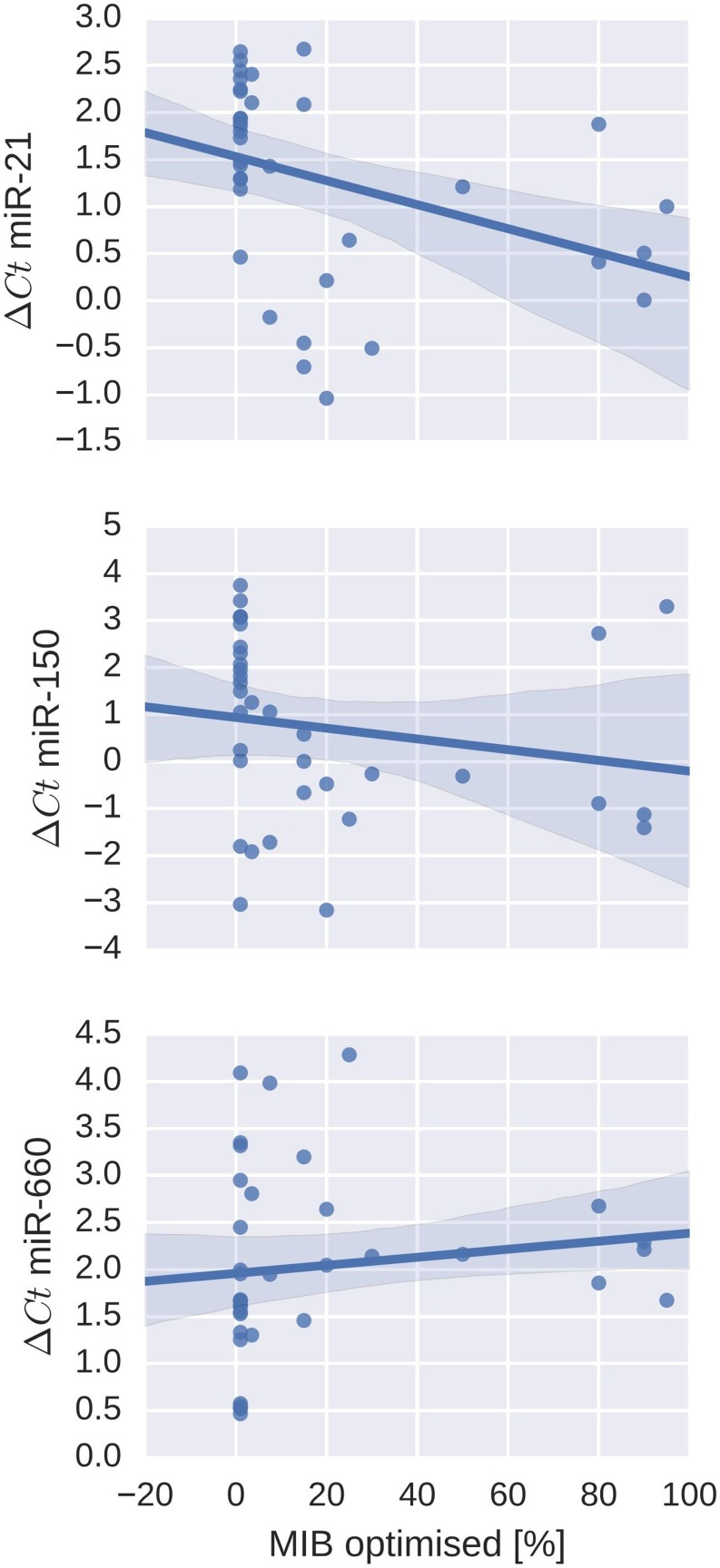
Correlation of miR-expression and proliferation indices (MiB1-staining) in cases with metastases Upper graph: ∆Ct values for miR-21 (*p* = 0.003; correlation coefficient -0.54). Middle graph: ∆Ct values for miR-150 (*p* = 0.026; correlation coefficient −0.43). Lower graph: ∆Ct values for miR-660 (*p* = 0.047; correlation coefficient 0.38). Lower ∆Ct values indicate higher miR expression and *vice versa*.

Overall, 7 miRs were differentially expressed in GEP-NEN with metastases (both nodal and distant) as compared to non-metastatic GEP-NEN: Expression of let-7b and miR-150 was significantly lower in metastatic disease (*p*-values 0.022 and 0.038) while miR-21, miR-30a-5p, miR-320, miR-331 and miR-660 expression was higher (*p*-values 0.038, 0.038, 0.038, 0.022 and 0.038, respectively; Figure 1A). Further analysis focusing exclusively on distant metastases (liver, peritoneum, spine and adrenal gland) revealed 5 miRs with higher expression in metastatic disease: miR-30a-5p, miR-210, miR-339-3p, miR-345 and miR-660 (all *p*-values between 0.0001 and 0.01; Figure 1B). Analysis focusing exclusively on nodal metastases revealed 32 differentially expressed miRs. 21 miRs were overexpressed (miR-15b, miR-17, miR-19a, miR-19b, miR-20a, miR-25, miR-106a, miR-106b, miR-135b, miR-185, miR-210, miR-331, miR-339-3p, miR-345 miR-374, miR-425-5p, miR-454, miR-484, miR-642, miR-660, miR-1243; all *p*-values between 0.0001 and 0.042; Figure 2) and 11 miRs were down-regulated (miR-26b, miR-30a-5p, miR-30e-3p, miR-125b, miR-127, miR-142-3p, miR-149, miR-150, miR-183#, miR-214, miR-1275; all *p*-values between < 0.0001 and 0.033; Figure 1C).

### Correlation of miR expression with Ki67 proliferation index

The proliferation index (as determined immunohistochemically by MiB1-staining) showed significant correlation with 44 different miRs. There was an inverse correlation with expression levels of 27 miRs (let-7e, let-7g, miR-103, miR-125a-5p, miR-126, miR-145, miR-16, miR-194, miR-199a-3p, miR-200b, miR-24, miR-27b, miR-28-3p, miR-29a, miR-30b, miR-324-3p, miR-324-5p, miR-342-3p, miR-375, miR-429, miR-484, miR-532, miR-532-3p, miR-574-3p, miR-642, miR-660 and miR-744; all *p*-values between < 0.001 and 0.049). Further, 17 miRs showed a positive correlation with the proliferation index (miR-106a, miR-106b, miR-10a, miR-10b, miR-135b, miR-142-3p, miR-146a, miR-150, miR-155, miR-17, miR-19b, miR-20a, miR-21, miR-222, miR-92a, miR-1274A and miR-93#; all *p*-values between < 0.001 and 0.049).

### Overlap between proliferation index and confirmed metastases

Comparison of miR expression profiles between cases with metastases and the proliferation index revealed an overlap for miR-150, miR-21 and miR-660 (Figure 2).

MiR-150 showed significantly lower expression in cases with metastases (*p* = 0.038) and a positive correlation with Mib1 staining (*p* = 0.026; correlation coefficient -0.43). MiR-21 and miR-660 both showed higher expression in cases with confirmed metastases (*p*-values 0.038 each), there was an inverse correlation with Mib1 staining for miR-660 (*p* = 0.047; correlation coefficient 0.38) and strong positive association with miR-21 (*p* = 0.003; correlation coefficient −0.54).

### miR profiles of GEP-NEN metastases and correlation with primary site

GEP-NEN metastases (both nodal and distant) arising from primary tumors located in the pancreas, ileum and ascending colon all showed several miRs differentially expressed specifically in these tissues. We found 13 miRs in metastases from ileal tumors, 7 in those from ascending colon and 5 in cases with pancreatic primaries. No such unique miR-expression patterns could be detected for metastases from the stomach. Tumors of the rectum and appendix were excluded from analysis due to small case numbers. A summary of dysregulated miRs and corresponding primary anatomic site of metastases is provided in Table 3.

**Table 3:** Overview of differentially expressed miRs of metastases dependent on their primary site

primary site of metastases	pancreas	ileum	ascending colon
overexpressed miRs	let-7b miR-127 miR-1274B miR-200c miR-720	let-7g miR-151-5p miR-194 miR-200a miR-200b miR-28-3p miR-30c miR-331 miR-375 miR-484 miR-744	miR-10b miR-135b miR-146a miR-19b miR-93#
down-regulated miRs		miR-345 miR-886-3p	miR-125a-5p miR-125b

Only miRs differentially expressed in specific anatomic site of primary of metastases are shown.

### Overlap between miR profiles of primary tumors and their metastases

**Figure 3:**
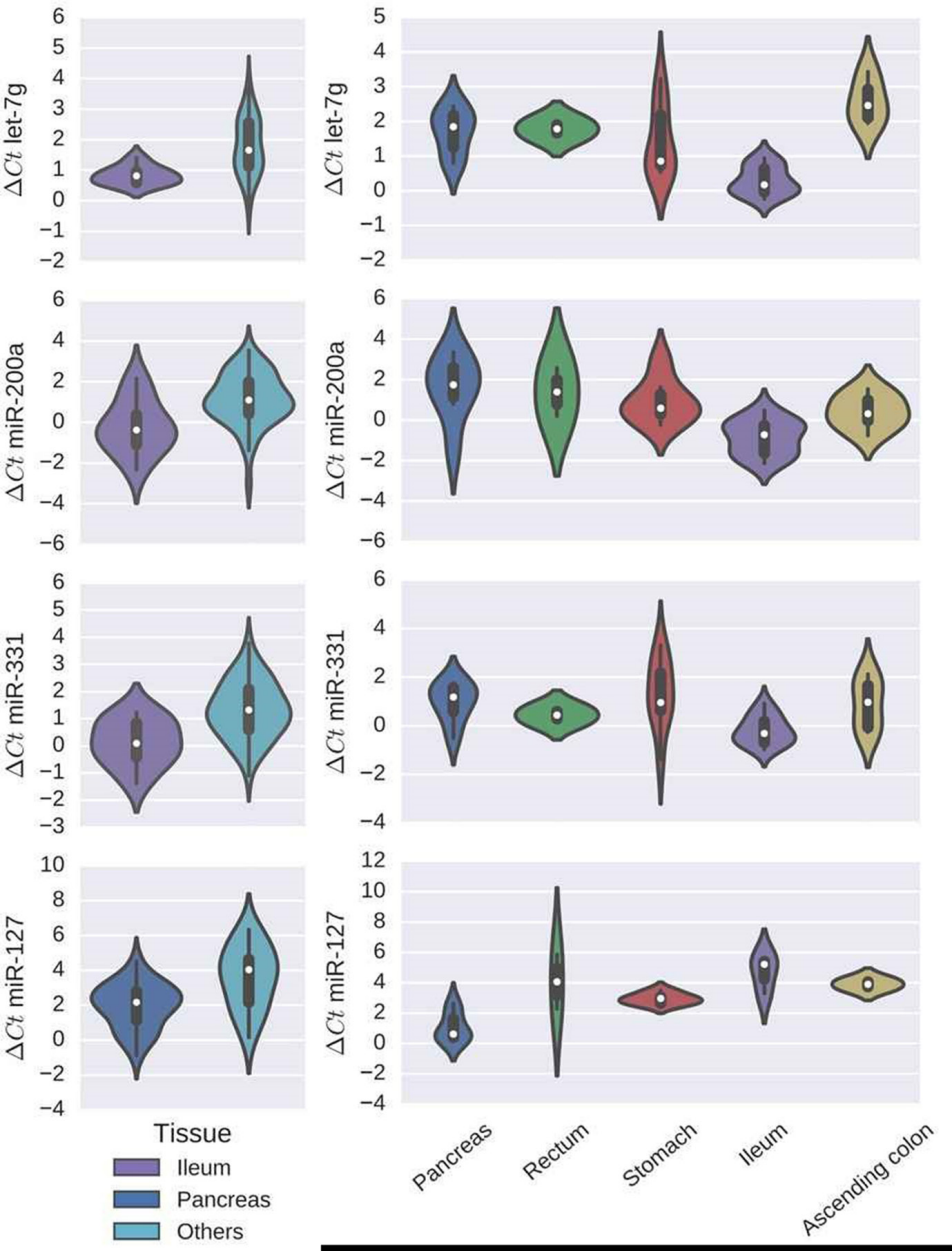
Overlap of miR-expression profiles between primary tumors and their metastases Left: Violin plots showing ∆Ct values for differentially expressed miRs in primary tumors. Green plots represent cases with ileal primary site, red plots represent cases with pancreatic primary site and blue plots all other primary locations. Right: Violin plots showing ∆Ct values of metastases according to specific primary site (blue = pancreatic primary, green = rectal primary, red = stomach primary, purple = ileal primary and yellow = primary tumor in ascending colon). The center of each violin holds a boxplot indicating the median (white dot). From left to right: First row: ∆Ct values for let-7g (primary tumor *p* < 0.0001, metastases *p* < 0.0001). Second row: ∆Ct values for miR-200a (primary tumor *p* = 0.029, metastases *p* = 0.0004). Third row: ∆Ct values for miR-331 (pt *p* = 0.0079, metastases *p* = 0.0013). Bottom row: ∆Ct values for miR-127 (primary tumor *p* = 0.014, metastases *p* = 0.006). Lower ∆Ct values indicate higher miR expression and vice versa.

Comparison of miR expression profiles between primary tumors and their metastases revealed the following overlap: In both pancreatic primaries and corresponding metastases miR-127 showed higher expression levels (*p*-values 0.014 and 0.006, respectively; Figure 3). Ileal primaries showed 3 overlapping miRs with their metastases with significantly higher expression for let-7g (*p*-values < 0.0001), miR-200a (*p*-values 0.029 and 0.0004, respectively) and miR-331 (*p*-values 0.0079 and 0.0013, respectively; Figure 3). No overlap was found for cases with primaries originating from the stomach or the ascending colon. Metastases from appendix and rectum NEN were excluded due to small numbers.

## DISCUSSION

In our study, we established miR profiles in a cohort of 76 GEP-NEN and 45 corresponding metastases to identify expression patterns for specific primary (anatomic) sites and their metastases (both nodal and distant). We also focused on the correlation between miR signatures of primary tumors and the proliferation index as a marker for aggressiveness. Additionally, we compared miR profiles in metastatic disease and localized disease. We identified specific miR patterns for primary tumors of pancreas, ileum, appendix and rectum as well as for metastases from pancreatic, ileal and ascending colon NEN. The identification of characteristic miR profiles in GEP-NEN metastases might lead to a major improvement in diagnostic pathways of CUP to further narrow down the most probable primary site.

Despite increasing awareness, data concerning miR expression in neuroendocrine tumors is still relatively scarce compared to various other tumor types such as adenocarcinomas or squamous cell carcinomas. Nevertheless, some studies focusing mostly on neuroendocrine tumors in the small bowel and pancreas have shown a multitude of dysregulated miRs (in comparison to adjacent normal tissue) including miRs generally implicated in cancer progression such as miR-19, miR-129-5p, miR-10b and miR-200 [12, 28]. However, a thorough comparison of different anatomic sites has not yet been attempted. Our study for the first time identified site-specific miR profiles in GEP-NEN, revealing several miRs differentially expressed specifically in pancreatic, ileal, rectal and appendiceal NEN (13 miRs, 9 miRs, 1 miR and 4 miRs, respectively). These included—for pancreatic primaries—only two miRs which have been previously reported to be also dysregulated in other malignant pancreatic tumors (adenocarcinomas: miR-126, miR-155) [29, 30]. However, in both instances, functions seem to differ: For miR-126, reduced expression resulting in KRAS elevation has been reported for pancreatic adenocarcinoma [29]; for miR-155, overexpression was associated with carcinoma progression, indicating a tumorigenic role [30] while miR-155 downregulation has also been reported (in pNEN) [22, 23]. This is, to some degree, in contrast with our data showing increased expression of miR-126 and decreased expression of miR-155 in pNEN.

Concerning ileal and rectal tumors, there was no overlap with other malignant tumors at the same anatomic site, although miR-16, miR-151 and miR-200c have been reported to be elevated also in inflammatory bowel disease (both Crohn’s disease and colitis ulcerosa) [31].

For primaries of the appendix, an overlap of differentially expressed miRs containing miR-200c and miR-223 has been shown with adenocarcinomas in the same region [32]. However, expression in adenocarcinomas was higher whereas in our series of appendix NEN expression levels were lower. Our data might possibly reflect site-specific differences in NEN. Yet, these profiles should be validated in larger series. It also seems that miR expression profiles do considerably vary according to tumor type, as several well-established miRs in carcinomas could not be detected in our NEN cohort.

Additionally, we further looked into specific miR profiles from different non-cancerous tissues using the “Human miRNA Tissue Atlas” [33]. Here it was reported that single miRs seemed to be tissue-specific, i.e. hsa-miR-205-5p, hsa-miR-514a-3p and hsa-miR-192-5p for large intestine/colon, hsa-miR-449c-5p and hsa-miR-449b-5p for small bowel. It was further concluded that -3p family seemed to be specific for pancreas while -5p family seemed to be specific for stomach. We then compared our miR findings of differentially expressed miRs in NEN to data provided in tissue atlas data bases and found several site-specific miRs in NEN (shown in Table 2) to correlate with already published data: hsa-miR-539-5p, hsa-miR-652-5p and hsa-miR-155-5p were found in both pancreatic NEN and normal pancreatic tissue; hsa-miR-200a-5p and hsa-miR-324-3p in ileal NEN and normal tissue of small intestine; hsa-miR-200c-3p, hsa-miR-24-3p and hsa-miR-151a-3p in colon and appendix [33, 34]. Unfortunately, up to date, studies focusing on miR expression in non-cancerous tissue are scarce and oftem done with only a handful of individuals and there sometimes seems to be a considerable overlap of miR expression in different tissues (especially brain, kidney, spleen and nerves) making most miRs not specific for defined anatomical sites.

Focusing on primaries with or without metastases in general, our study confirms earlier results from investigations focusing on miRs in plasma of patients with small intestine NEN: Primaries with metastatic disease showed a higher expression of miR-21 and miR-600 and a lower expression of miR-150 [35]. Additionally, miR-21 has also been reported to be commonly associated with liver metastases in pancreatic neoplasias [36], probably indicating a more aggressive tumor biology and a worse clinical outcome. In line, these 3 miRs (miR-21, miR-150 and miR-660) also showed a strong and concordant association with the proliferation index (Ki67). We additionally found 41other miRs of either an inverse or positive correlation with the proliferation index: We recently reported a correlation between miR-642 and proliferation index in a cohort of 37 patients with pNEN [27]. Further, high miR-155, miR-221 and miR-222 expression has been reported to show a correlation with higher proliferation index in pancreatic carcinomas [30, 36, 37].

Overall, we were able to validate some previously reported associations between miRs and tumor proliferation and moreover identified additional miRs associated with Ki67 index. These new miRs could potentially be used as additional markers for proliferation rate in further studies, especially in cases where mitotic counts are intermediate between two grading groups.

After establishing miR profiles for metastatic cases in general, we aimed to subdivide cases into nodal (regional) and visceral (distant) metastases. We found 5 and 32 miRs differentially expressed in cases with distant and nodal metastases, respectively. This discrepancy might be due to the fact that 56 patients showed nodal metastases while only 19 had evidence of distant metastases. In line with previously published data, we confirmed overexpression of both miR-19a and miR-19b in cases with lymph node metastases [17–20, 25]. There was no overlap between our data and other miRs in the literature, which focused mainly on metastatic ileal NEN (lower expression of miR-1, miR-10b, miR-129-5p, miR-133a, miR-143-3p, miR-145, miR-146, miR-215, miR-222 and miR-31; overexpression of miR-183, miR-19a, miR-19b, miR-200a and miR-488). Some other miRs (i.e. miR-10b and miR-17) have been reported to be associated with invasiveness, tumor dissemination and generally worse survival in pancreatic cancer [38, 39], although a direct link to metastases has not yet been established. Yet others (miR-125b) have been reported to be associated directly with the potential for lymph node metastases in other malignant tumors such as colorectal and gastric carcinomas [40, 41].

We demonstrated overlaps with several miRs that have been reported to show an association with lymph node metastases and additionally detected multiple miRs indicating either lymph node or distant metastases. Whether these might be clinically relevant for patients and predictive for the course of the disease needs to be verified in further studies.

Further, we identified specific miR profiles of GEP-NEN metastases arising from primary tumors located in the pancreas, ileum and ascending colon, revealing several dysregulated miRs for each organ (5 miRs, 13 miRs and 7 miRs, respectively). To date, there is no comparable study that focused on differences in miR profiles in GEP-NEN metastases from differing primary sites. Only one study reported on the differences between small bowel NEN and their metastases, identifying 5 dysregulated miRs (overexpression of miR-204-5p, miR-7-5p and miR-375 in metastases as well as lower expression of miR-1 and miR-143-3p) with only 1 overlap (miR-375 in metastases from ileal primaries)compared to our results [25]. However, as there are no further comparable studies, especially for pancreatic tumors, which constitute the largest proportion of our series, it remains to be seen whether these signatures and patterns can be further replicated.

Following these observations, we tried to establish a possible overlap between miR signatures in primary tumors and corresponding metastases which also has not been attempted before. There was 1 overlapping miR in pNEN (miR-127) and 3 in ileal NEN (let-7g, miR-200a and miR-331). These results might allow a classification of metastases from unknown primary tumors to predict the most probable primary location for some GEP-NEN. Due to small numbers of stomach, rectum and appendix NEN in our series—for now—no conclusion can be drawn for these tumor entities. However, in CUP-NEN, where the primary cannot be found even after a thorough search, our results in concert with further molecular or immunohistochemical analyses [9] could help clinicians to divide CUP cases in diagnostic groups.

In conclusion, our study conducted, for the first time, a thorough analysis of GEP-NEN and their corresponding metastases. We did not only determine site-specific miR profiles and patterns for primary tumors, but also demonstrated their association with proliferation indices, thereby trying to establish a link between metastases and their most probable site of origin. However, as this study represents exploratory work, caution should be taken regarding too strong conclusions and further investigations are necessary to validate results.

## MATERIALS AND METHODS

### Selection of cases

Samples from formalin-fixed, paraffin-embedded (FFPE) tissue containing GEP-NEN primary tumors and metastases were included in the present study. All cases were collected as part of routine clinical care at the University Hospital of Schleswig-Holstein, Campus Luebeck during 1993–2014. All analyses performed were in accordance with the Declaration of Helsinki and had been approved by the local Ethics Committee beforehand (reference number 13–093).

### Histologic examination

Samples were carefully examined by two researchers (CT, NZ) with a light microscope (Axioskop, Zeiss, Jena, Germany) and diagnosis was confirmed using haematoxylin-eosin-, chromogranin A- and synaptophysin-stained slides. Proliferation indices were determined immunohistochemically with MiB1-staining (Dako, Hamburg, Germany, clone MiB-1, dilution 1:100) in accordance with the current WHO classification (≤ 2% for grade 1; 3–20% for grade 2 and > 20% for grade 3).

### RNA isolation and microRNA profiling

RNA for profiling of miR was isolated from FFPE-tissue using the RecoverAll™ total nucleic acid isolation kit (Applied Biosystems, Carlsbad, California, USA). RNA concentrations were quantified using the NanoDrop Spectrophotometer (NanoDrop Technologies, Montchanin, New Castle, Delaware, USA). Afterwards, reverse transcription (RT) using amounts of 350 ng of total RNA in 3 µl aqua dest. 3.7 µl Master Mix of the „TaqMan^®^ MicroRNA Reverse Transcription Kit“ combined with „Megaplex RT Primer, human pool A v2.1“ or „Megaplex RT Primer, human pool B v3.0“ (Applied Biosystems, Carlsbad, California, USA). In this step the miR is transcripted into complementary DNA (cDNA) and amplified using the Thermocycler (Biometra, Göttingen, Germany). Thereafter 5.3 µl of the RT-products were combined with PCR Master Mix and nuclease-free water from the „TaqMan^®^ Universal PCR Master Mix, no AmpErase^®^ UNG“ (Applied Biosystems, Carlsbad, California, USA). Each reservoir of the „TaqMan^®^ Array Human MicroRNA A+B Cards Set v3.0“ (Applied Biosystems, Carlsbad, California, USA) was filled with 100 µl of the PCR Master mix-cDNA mix and was centrifuged using „HeraeusMegafuge™” (ThermoFisher Scientific, Waltham, Massachusetts, USA) and the cards were sealed by using „TaqMan^®^ Array Micro Fluidic Card Sealer“ (Applied Biosystems, Carlsbad, California, USA). Finally real time quantitative PCR (qPCR) was performed by using the „TaqMan 7900HT Fast Real-Time PCR System“ and „SDS Software v2.2.2“ (Applied Biosystems, Carlsbad, California, USA) to obtain raw cycle threshold (Ct) values. All reactions were carried out according to the manufacturer’s instructions. All data was transformed into Excel for further assessment. ***∆***Ct values were used to determine the amount of miR in a sample (both parameters showing an inverse correlation).

### Data processing and statistical analysis

Many miRs contained in the assay could only be detected in few samples. In order to focus on the most reliable data, any Ct values greater or equal to 32 were discarded, as recommended by the manufacturer of the miR measurement assay. Additionally, we excluded any miR with less than 40 valid values (Ct < 32). Thus, all analyses were conducted on 83 miRs measured in at least 40 out of 76 samples. All data was normalized to the lower-quartile per sample. Normalized Ct value distributions per miR were visually checked for near-normality and variances within different groups were visually checked for homogeneity. Comparisons between multiple groups (e.g., different tissues of origin) were performed with an ANOVA followed by a non-parametric Dunn post-hoc test. Binary comparisons (e.g., primary tumors vs. metastases) relied on Welch’s *t*-test. Correlation coefficients were determined by Spearman’s rho. All *p*-values were corrected for multiple testing employing the Benjamini-Hochberg procedure with a conventional target FDR of 5% considering all tests actually performed. Results were deemed significant if the Benjamini-Hochberg-corrected *p*-value was smaller or equal to 0.05. All statistical analyses have been implemented in python (v2.7; www.python.org) using packages from the anaconda distribution (www.anaconda.org). Scripts are available upon request.
